# Sleep Disturbance and Atopic Dermatitis: A Bidirectional Relationship With Clinical and Therapeutic Implications

**DOI:** 10.7759/cureus.112165

**Published:** 2026-07-06

**Authors:** Iwona Kaliciak, Oliwia Korda

**Affiliations:** 1 Department of Medicine, University Clinical Hospital, Poznan, POL

**Keywords:** atopic dermatitis, circadian rhythm, insomnia, melatonin, pruritus, quality of life, skin barrier, sleep disturbance

## Abstract

Sleep disturbance is one of the most frequent and clinically consequential problems in atopic dermatitis. Current evidence indicates that it is not merely a secondary symptom of pruritus but a complex disease domain associated with higher inflammatory activity, sleep fragmentation, impaired quality of life, daytime fatigue, and anxiety or depressive symptoms. The best established pathway runs from active dermatitis to impaired sleep: nocturnal itch, scratching, pain, xerosis, heat sensation, and epidermal barrier dysfunction promote repeated awakenings and difficulty maintaining sleep. However, growing mechanistic and epidemiological evidence supports a bidirectional model. Insufficient, fragmented, or circadian-misaligned sleep may impair barrier repair, increase transepidermal water loss, alter neuroimmune itch signaling, and perpetuate behavioral amplification through scratching, stress, and reduced treatment adherence. This narrative review synthesizes recent literature on the epidemiology, sleep phenotype, biological mechanisms, assessment tools, therapeutic evidence, and research gaps linking sleep disturbance with aggravation of atopic dermatitis. The clinical implication is practical: sleep should be assessed as a core treatment outcome in atopic dermatitis, and persistent sleep disruption should be interpreted as a marker of undercontrolled disease and a potential amplifier of cutaneous inflammation.

## Introduction and background

Atopic dermatitis is a chronic, relapsing inflammatory skin disease characterized by epidermal barrier dysfunction, type 2 immune activation, xerosis, and persistent pruritus [1-3]. Sleep disturbance has traditionally been described as a consequence of itch, but the last decade of research has reframed sleep as a clinically important disease domain [1,2,4]. Pediatric reviews, population-based adult studies, actigraphy and polysomnography studies, psychometric validation papers, and interventional trials all show that disturbed sleep is common, measurable, and closely linked with disease severity and daily functioning [1,4-6].

Atopic dermatitis disrupts sleep in children and adults, and this relationship is supported by epidemiological studies, objective sleep studies, and systematic reviews [4,6-12]. The more clinically relevant question is whether sleep disturbance can also aggravate atopic dermatitis and contribute to a self-reinforcing cycle of itch, scratching, impaired barrier recovery, inflammation, stress, and further sleep fragmentation [1,3,5,13,14]. This review, therefore, focuses on sleep disturbance not only as a symptom, but as a potential disease-modifying factor in atopic dermatitis [1,5,13,14].

## Review

Methods of literature synthesis

This narrative review was prepared using literature retrieved from PubMed, PubMed Central, Scopus, Google Scholar, and publisher websites. Additional sources were identified through manual screening of reference lists from relevant review articles and original studies. The search focused primarily on publications from January 2016 to June 2026, with priority given to recent evidence on sleep disturbance, insomnia, pruritus, circadian regulation, epidermal barrier dysfunction, patient-reported outcomes, and treatment-related sleep improvement in atopic dermatitis.

The following keywords and Medical Subject Headings terms were used individually and in combination: "atopic dermatitis", "eczema", "sleep disturbance", "insomnia", "sleep quality", "sleep disorders", "actigraphy", "polysomnography", "wake after sleep onset", "nocturnal pruritus", "itch", "skin barrier", "transepidermal water loss", "circadian rhythm", "melatonin", "interleukin 31", "quality of life", "patient-reported outcomes", "dupilumab", "abrocitinib", "upadacitinib", "crisaborole", "nemolizumab", and "cyclosporine". Boolean operators "AND" and "OR" were applied to refine the search strategy.

Eligible sources included narrative reviews, systematic reviews, meta-analyses, observational studies, cohort studies, randomized trials, psychometric validation studies, mechanistic studies, and clinically relevant interventional reports related to dermatology, allergy and immunology, sleep medicine, circadian biology, neuroimmune itch, skin barrier function, and atopic dermatitis therapy. English-language, peer-reviewed publications and full-text articles were prioritized. Studies were excluded when they focused exclusively on dermatologic diseases other than atopic dermatitis, cosmetic sleep-related interventions, non-peer-reviewed commentary, or outcomes unrelated to sleep disturbance, itch, barrier function, quality of life, or atopic dermatitis severity.

Epidemiology of sleep disturbance in atopic dermatitis

Sleep disturbance in atopic dermatitis is heterogeneous [2,4,10]. It includes difficulty initiating sleep, nocturnal awakenings, prolonged wake after sleep onset, reduced sleep efficiency, early morning awakening, daytime sleepiness, fatigue, restless sleep, nocturnal scratching, caregiver sleep disruption, and, in selected patients, comorbid primary sleep disorders [2,4,6,7,9-12,15]. Across studies, the dominant phenotype is often sleep-maintenance insomnia rather than a large reduction in total sleep time [7,8,11,12].

Adult population data demonstrate a substantial burden [6,10]. Yu et al. reported that adults with atopic dermatitis had higher adjusted odds of shorter sleep duration, difficulty falling asleep, early awakening, and fatigue-related impairment in instrumental activities of daily living [6]. A later US adult survey found that 40.7% of adults with atopic dermatitis had at least one night of disease-related sleep disturbance in the previous week, while 79.7% reported some trouble sleeping during the previous three days [10]. Sleep disturbance also correlated with higher Patient-Oriented Eczema Measure scores, worse Dermatology Life Quality Index scores, pain, itch, and anxiety or depressive symptoms [10].

Pediatric evidence is similarly strong [7-9]. In a case-control actigraphy study of children with moderate-to-severe atopic dermatitis, wake after sleep onset was approximately twice that of matched controls, and objective disease severity correlated with wake after sleep onset [7]. A longitudinal birth cohort analysis found that active atopic dermatitis was associated with 48% higher adjusted odds of impaired sleep quality across childhood and adolescence, despite a clinically minimal mean difference in sleep duration [8]. In a weighted US pediatric sample, sleep disturbance was estimated in 66.9% of school-aged children with atopic dermatitis, with severe sleep disturbance much more likely in moderate or severe disease than in mild disease [9].

Meta-analytic data sharpen this picture [11,12]. A meta-analysis of objective sleep studies found that, compared with controls, patients with atopic dermatitis had less total sleep time, lower sleep efficiency, longer wake after sleep onset, and longer rapid eye movement latency [11]. A 2025 meta-analysis estimated the pooled prevalence of sleep disorders in atopic dermatitis at 43.4%, with high rates of insomnia and nocturnal awakening phenotypes [12]. These findings support routine sleep screening as part of standard disease assessment [2,4,10,12].

Clinical sleep phenotype: fragmentation more than duration

One clinically important lesson from recent studies is that sleep duration alone is an incomplete marker of sleep burden in atopic dermatitis [7,8,11]. Patients may report severe sleep impairment while showing only modest average reductions in total sleep time [8,11]. Fragmentation, repeated arousals, scratching episodes, and prolonged wake after sleep onset are often more relevant to daytime fatigue and quality of life than the total number of hours spent in bed [7,8,11].

This distinction matters for both clinical care and research because sleep continuity and sleep-related impairment are validated patient-centered domains in atopic dermatitis [16-19]. A patient who wakes repeatedly because of an itch may experience nonrestorative sleep, impaired concentration, irritability, and reduced adherence to complex topical regimens [2-4,10]. Similarly, caregivers of children with atopic dermatitis may experience substantial sleep loss and psychosocial strain even when the child's total sleep time appears superficially acceptable [9,20].

Mechanisms linking atopic dermatitis to sleep loss

The best supported direction is from active dermatitis to sleep disturbance [4,6-12]. Nocturnal itch, scratching, pain, dryness, heat sensation, sweating, and inflamed skin create repeated arousals that reduce sleep continuity [1,2,4,7,21]. Pruritus remains central, but it is not the only determinant [2,4,7,21]. The relatively imperfect correlation between simple itch ratings and objective sleep disruption suggests that barrier status, inflammatory activity, central arousal, mood, and environmental triggers also contribute [7,21,22].

Neuroimmune signaling provides a biological explanation for the strong link between itch and sleep fragmentation [1-3,23,24]. Interleukin 31 signaling, type 2 inflammation, and related pruritogenic inflammatory mediators are implicated in chronic itch in atopic dermatitis [1-3,23,24]. This pathway is especially important at night, when itch can trigger arousal, scratching, further barrier injury, and renewed inflammation [1-4,7]. The rapid improvement in itch and sleep observed with interleukin 31 pathway targeting supports the clinical relevance of this mechanism [23,24].

Circadian biology is another major pathway [1,13]. Healthy skin shows rhythmic changes in temperature, barrier recovery, water loss, immune activity, and cutaneous blood flow [1,13]. In atopic dermatitis, these rhythms may become maladaptive [1,13]. A controlled physiologic study found that barrier function in atopic dermatitis worsened in the evening, a time when healthy skin would typically show recovery [13]. This observation is consistent with a model in which evening barrier vulnerability amplifies nocturnal itch and sleep maintenance insomnia [1,13].

Can poor sleep aggravate atopic dermatitis?

The reverse pathway, in which sleep disturbance worsens atopic dermatitis, is less mature than the dermatitis-to-sleep pathway, but it is biologically plausible and increasingly supported [1,5,13,14]. Sleep deprivation and circadian misalignment can impair barrier recovery, alter skin hydration, increase transepidermal water loss, and promote inflammatory activation [1,3,13]. Because atopic dermatitis is fundamentally a barrier-vulnerable and immune-reactive disease, any process that reduces epidermal repair may plausibly increase flare susceptibility [1-3,13].

Melatonin provides a bridge among sleep, circadian timing, and inflammation [1,5]. A randomized crossover trial in children with atopic dermatitis showed that 3 mg of melatonin nightly for four weeks shortened sleep-onset latency and improved Scoring Atopic Dermatitis (SCORAD) compared with placebo [5]. The improvement in eczema severity did not simply track with sleep-latency change, raising the possibility that melatonin may act through combined sleep-promoting, circadian, antioxidant, or anti-inflammatory mechanisms [1,5].

Genetic epidemiology adds early support for bidirectionality [14]. A Mendelian-randomization study reported that genetically predicted insomnia was associated with a higher risk of atopic dermatitis [14]. This finding does not prove that treating insomnia will reduce established dermatitis activity in routine care, but it strengthens the case that poor sleep is not only a passive consequence of eczema [14].

Psychological and social amplifiers

Sleep disturbance in atopic dermatitis is partly cutaneous, partly neuroimmune, and partly psychosocial [2,3,10]. Adults with worse sleep disturbance report poorer health-related quality of life and more anxiety or depressive symptoms [10]. Children may experience fatigue, inattention, irritability, and family-level sleep disruption [9,20]. The relationship is likely bidirectional: active disease worsens sleep and mood, while poor sleep and stress increase itch perception, reduce coping capacity, and may decrease adherence to emollients or topical anti-inflammatory therapy [2,3,10,25].

Social context also modifies risk [8-10]. Studies have linked worse sleep outcomes with disease severity, comorbid asthma or allergic rhinitis, lower household income, and other markers of vulnerability [8-10]. These modifiers matter because they identify patients in whom sleep disturbance may persist despite apparently reasonable skin-directed therapy [8-10]. In such patients, assessment should include mental health symptoms, sleep environment, caregiver burden, and possible primary sleep disorders [2,4,10,15].

Assessment of sleep in clinical practice and trials

Recent psychometric work has improved the measurement of sleep in atopic dermatitis [16-19,26]. Validated tools include the Patient Reported Outcomes Measurement Information System (PROMIS) Sleep Disturbance and Sleep-Related Impairment measures, sleep numerical rating scales, single-item patient-reported sleep assessments, and sleep diaries designed for moderate-to-severe atopic dermatitis [16-19,26]. These instruments are important because they capture sleep impact beyond duration, including nocturnal awakenings, sleep quality, daytime impairment, and daily symptom variation [16-19,26].

In routine practice, the minimum useful sleep history should ask about sleep-onset difficulty, number of awakenings, wake after sleep onset, nocturnal itch and pain, scratching, daytime sleepiness, fatigue, school or work impairment, caregiver sleep, and symptoms suggesting obstructive sleep apnea or another primary sleep disorder [2,4,10-12,15]. Actigraphy and polysomnography are most useful when symptoms are severe, when reports and clinical signs are discordant, or when comorbid sleep disorders are suspected [15,27].

Therapeutic implications

The most reliable way to improve sleep in atopic dermatitis is to control cutaneous inflammation and nocturnal itch [2,4,20,22,24,28-30]. Cyclosporine improved several objective nocturnal parameters in adults with moderate-to-severe disease [28]. Pooled dupilumab trial analyses showed significant sleep improvement in adults [22]. Abrocitinib and upadacitinib analyses reported rapid patient-reported improvements in symptom burden, nighttime itch, and sleep-related outcomes [29,30]. Pediatric crisaborole analyses showed reductions in child sleep disruption and family sleep burden [3].

The interleukin 31 pathway is particularly relevant because it directly links immune activity, itch, and sleep [3,23,24]. Nemolizumab phase 3 analyses reported very rapid improvement of itch and sleep disturbance in atopic dermatitis and prurigo nodularis populations [24]. Earlier anti-interleukin-31 receptor work also supports the concept that targeting neuroimmune itch can produce clinically meaningful sleep benefit [23].

Sleep-targeted treatment is less developed but clinically important [2,4,5,25]. The strongest direct evidence remains pediatric melatonin, which improved both sleep-onset latency and SCORAD in a randomized trial [5]. Behavioral strategies are widely recommended because they are low risk: consistent sleep and wake times, a cool and dark bedroom, avoidance of late caffeine and evening screen exposure, nonirritating bedding and clothing, short nails, stress reduction, and treatment of anxiety or depressive symptoms [2,4]. Reviews emphasize that evidence for sleep-specific adjunctive interventions remains limited, while digital sleep-scratch monitoring has preliminary interventional support [2,4,25].

Research gaps and future directions

The main limitation of the current literature is causal design. Cross-sectional studies establish burden and association, but they do not fully determine directionality. Longitudinal pediatric studies and Mendelian-randomization data strengthen the bidirectional model, yet the field still lacks trials designed to answer the most important clinical question: if sleep improves independently, does atopic dermatitis activity decrease?

A second gap is measurement heterogeneity. Studies have used sleep duration, single sleep questions, PROMIS measures, numerical rating scales, sleep diaries, actigraphy, polysomnography, and nighttime itch scales across different recall windows. Wider adoption of validated sleep-specific endpoints would improve comparability across trials and registries [17-19,26].

A third gap is mechanistic integration. Future studies should combine objective sleep monitoring, itch tracking, barrier metrics such as transepidermal water loss, circadian biomarkers, microbiome sequencing, inflammatory markers, and patient-reported outcomes within the same cohort. Such designs would help determine whether poor sleep directly aggravates epidermal dysfunction or mainly reflects uncontrolled inflammation.

## Conclusions

Sleep disturbance is a major and clinically meaningful component of atopic dermatitis. The strongest evidence confirms that active dermatitis, nocturnal itch, scratching, barrier dysfunction, and type 2 inflammation disrupt sleep, especially through sleep fragmentation and wake after sleep onset. At the same time, mechanistic and emerging causal evidence suggest that poor sleep may contribute to worsening disease activity in atopic dermatitis by impairing barrier repair, increasing physiological vulnerability, intensifying neuroimmune itch, and worsening stress-related behavioral loops.

For clinical practice, sleep should be treated as a core outcome rather than a secondary quality-of-life detail. Persistent sleep disturbance should prompt reassessment of disease control, nocturnal itch, adherence, mental health, sleep hygiene, and possible comorbid sleep disorders. For research, the next priority is to test whether sleep-targeted interventions can reduce dermatitis activity beyond the benefits achieved by modern anti-inflammatory therapy. Until then, the most balanced conclusion is that sleep disturbance is both a marker of undercontrolled disease and a plausible amplifier of the atopic dermatitis inflammatory cycle.
